# Surveillance, response systems, and evidence updates on emerging zoonoses: the role of one health

**DOI:** 10.3402/iee.v3i0.21386

**Published:** 2013-12-13

**Authors:** G. V. Asokan, Ramanathan K. Kasimanickam, Vanitha Asokan

**Affiliations:** 1 Head, Public Health Program, College of Health Sciences, University of Bahrain, Sakhir, Kingdom of Bahrain; 2 Associate Professor, Department of Veterinary Clinical Sciences, College of Veterinary Medicine, Washington State University, Pullman, WA, USA; 3 Head of Pediatrics, American Mission Hospital, Saar, Bahrain, Kingdom of Bahrain

**Keywords:** surveillance, evidence updates, emerging zoonoses, one health

## Abstract

Globally, emerging zoonotic diseases are increasing. Existing surveillance systems for zoonoses have substantial gaps, especially in developing countries, and the systems in place in the developed world require improvements. Resources and updates on evidence-based practice (EBP) for zoonoses are sparser in the veterinary literature as compared to the medical literature. Evidence updates for emerging zoonoses are either absent or rudimentary in both human and veterinary medicine. A ‘one-health’ concept, including a global signaling surveillance system for emerging zoonoses, will be essential for correct diagnoses, interventions, and public health strategies. An open access EBP platform supported by builders of EBP resources is urgently needed to counter emerging zoonoses.

The World Health Organization (WHO) defines emerging zoonosis as ‘a zoonosis that is newly recognized or newly evolved, or that has occurred previously but shows an increase in incidence or expansion in geographical, host or vector range’ (1). The emergence of a zoonosis can result from different factors, including global warming and climate change; anthropogenic factors such as trade, tourism, deforestation, changed agricultural practices and livestock farming, and hunting; and natural factors such as pathogen adaptation and animal migration (2). Emerging zoonotic diseases are increasing, causing severe economic impacts and animal and human health risks. The World Bank estimates that zoonotic disease outbreaks – mostly of emerging zoonoses – have caused global losses exceeding $US200 billion, including reduced trade, tourism, and tax revenues in the past 10 years (3). Among the infectious agents considered to be emerging, 175 species from 96 genera have been documented, of which 132 (75%) species from 78 genera are zoonotic. It has been estimated that zoonotic pathogens are twice more likely to be emerging than their non-zoonotic counterparts (4).

There is a large and complex interdependence between different species on our planet. Therefore, it is essential to combine disciplines in biology, veterinary medicine, and human medicine in order to understand and control emerging diseases (5). The management of emerging zoonoses is outside the scope of traditional medicine (bedside practice) and veterinary medicine (stall side practice); consequently, there has been an enhanced collaborative effort between the two professions, resulting in the new ‘one-health’ concept. This is a systems-approach concept which consolidates the ‘collaborative effort of multiple disciplines working locally, nationally, and globally to attain optimal health for people, animals and our environment’ (6). However, decision making for emerging zoonoses is an enormous challenge as there are large uncertainties and a lack of data. In that regard, medical and veterinary practitioners need timely, relevant, reliable evidence and current, integrated knowledge to manage emerging zoonoses.

## Existing surveillance and response systems

There are two general surveillance systems for early warning and preparedness: (A) ‘Syndromic surveillance’ that focuses on disease trends by analyzing data on a cluster of clinical symptoms potentially associated with a disease or a phenomenon (in the absence of pathogen identification); and (B) ‘Risk surveillance’ that focuses on detecting risk factors for disease transmission without estimating the prevalence of pathogens, or identifying clinical features. Even in developed countries, syndromic surveillance for animal diseases, including zoonoses, are rudimentary. To overcome this deficiency, the Triple-S project, co-funded by the European Commission, is forging synergies between the human and animal health sectors by providing scientific and technical guidance on syndromic surveillance in real-time or near real-time which encompasses emerging zoonoses (7). Examples of international organizations that report and respond to zoonoses, including emerging zoonoses, are listed in Table 1. These organizations, including the WHO, the World Organization for Animal Health (OIE), and the Food and Agricultural Organization (FAO) have several different roles, including monitoring, notification regarding cases/and outbreaks, and technical support.

**Table 1:** International outbreak and response systems

Outbreak response system	International organization/society	Scope of work	URL
GLEWS – Global Early Warning System for Major Animal Diseases, including Zoonoses	FAO OIE WHO	Alert and response mechanisms for major animal diseases, including zoonoses	http://www.glews.net/
WAHID – World Animal Health Information System and Database – Interface	OIE	Notification of cases of the main animal diseases, including zoonoses, and subsequent analyses of these data	http://www.oie.int/wahis_2/public/wahid.php/Wahidhome/Home
GOARN – Global Outbreak Alert and Response Network	WHO	Quick and appropriate technical support to populations affected by human disease epidemics, including zoonoses, on a national, regional, or even international level	http://www.who.int/csr/outbreaknetwork/en/
OFFLU – Joint Network of Expertise on Animal Influenza	FAO OIE	Monitor and control infections of avian influenza	http://www.offlu.net/
EMPRES-i-Emergency Prevention System for Transboundary Animal and Plant Pests and Diseases Information System – Global Animal Disease Information System	FAO	Early warning and response to transboundary animal diseases (TADs), including emerging zoonoses	http://empres-i.fao.org/eipws3g/#h=0
ProMED-mail – Program for Monitoring Emerging Diseases	International Society for Infectious Diseases – ISID	Internet-based reporting system dedicated to rapid global dissemination of information on outbreaks of infectious diseases and acute exposures to toxins that affect human health, including those in animals and in plants grown for food or animal feed	http://www.promedmail.org/
Weekly Epidemiological Record (WER)	WHO	Disseminates epidemiological information on cases and outbreaks of diseases under the International Health Regulations and on other communicable diseases of public health importance, including emerging or re-emerging infections	http://www.who.int/wer/2013/wer8801/en/index.html

Other than the country-specific surveillance systems, certain global, region-specific, and non-governmental organizations which provide information related to zoonoses in general are:

1. Morbidity and Mortality Weekly Report (MMWR) of CDC
2. Global Public Health Information Network (GPHIN) of Health Canada
3. UK public health network for zoonoses
4. European Centre for Disease Prevention and Control (ECDCMedVetNet and PACNET in the Pacific region)
5. Sentiweb in France
6. Mekong Basin Disease Surveillance Network (MBDS) – China, Cambodia, Vietnam, Thailand, Myanmar, and Lao PDR
7. Middle East Consortium on Infectious Disease Surveillance (MECIDS) – Israel, Jordan, and the Palestinian Territory
8. South-eastern European Health Network (SEEHN) – Albania, Bosnia and Herzegovina, Bulgaria, Croatia, Montenegro, the Republic of Moldova, Romania, Serbia, and the former Yugoslav Republic of Macedonia (FYROM)
9. Asian Partnership on Emerging Infectious Disease Research (APIER) – Cambodia, China, Lao PDR, Indonesia, Thailand, and Vietnam
10. South African Centre for Infectious Disease Surveillance (SACIDS) – DR Congo, Mozambique, South Africa, Zambia, and Tanzania
11. East African Integrated Disease Surveillance Network (EAIDSNet) – Kenya, Tanzania, and Uganda
12. Non-governmental organizations, including the Red Cross, Red Crescent, and so on

Although there are early warning and surveillance systems in the developed world for timely recognition of emerging zoonotic diseases in humans and farm animals, improvements are needed. However, detection and surveillance systems for emerging zoonoses in the developing world are much less functional. In that regard, surveillance systems for wildlife, exotic animals, and companion animals have not been established. Wildlife represents a significantly large and often unknown reservoir for emerging zoonoses, in addition to being a reservoir for re-emergence of zoonoses previously controlled. Factors for emergence of zoonoses from wildlife include a burgeoning human population leading to exploitation of forest areas for agriculture and dwelling, ecotourism, outdoor activities, and live animal trade (8).

## Evidence-based practice

Evidence-based practice (EBP) – an interdisciplinary approach to clinical practice – in human medicine has evolved over the past two decades due to a parallel progressive transformation in the following core domains: clinical trial registries, research reporting standards, systematic reviews, collaborations that produce and archive systematic reviews, MEDLINE indexing, and evidence update resources for point-of-care decision support. In contrast, the information and infrastructure supporting evidence-based veterinary medical practice in all of the above elements are less developed due to less public funding being made available to the veterinary side as compared to human health care (9).

The EBP resources include websites that provide processed information on best available research evidence which are critically appraised, integrated, concisely summarized, and regularly updated as new research evidence becomes available. Distilling and disseminating evidence, a process of knowledge translation, which evaluates relevance and applicability in various scenarios, is made accessible to health providers and those making policy decisions. These EBP resources are either freely accessible or via subscription. Access to such resources addresses several barriers identified by health care decision makers regarding access to evidence-based information, including limitations of time and lack of knowledge regarding resources that synthesize, summarize, and assess evidence for quality. Some of the available resources that provide ready access to high-quality, pre-processed research evidence include Cochrane database, National Guideline Clearinghouse (NGC-USA), American College of Physicians–American Society of Internal Medicine (ACP Journal Club), National Institute for Health and Clinical Evidence (NICE – UK), Bandolier, Clinical Evidence, Database of Abstracts of Reviews of Effectiveness (DARE). Meta search sources include the Turning Research Into Practice (TRIP) Database, The British Medical Journal (BMJ) evidence centre, SUM Search, and other noteworthy resources, including OVID, Clinical Queries in PubMed, NHS Centre for Reviews and Dissemination (CRD) Databases, and Public Health plus. Some of these have been assessed using quality, relevance, and newsworthiness filters and are regularly updated.

The above listed EBP resources provide evidence updates on etiology, diagnosis, treatment, prognosis, and economics but are restricted to medical conditions only. Unfortunately, there are apparently no corresponding open, related guideline repositories or registries in veterinary medical practice, although the Evidence-Based Clinical Practice Guidelines of agency for health research and quality (AHRQ) has a short index for zoonoses-related guidelines (10).

## The essentials of evidence updates on one health

The focus of this commentary is on resources for evidence updates to counter emerging zoonoses, for example, Severe Acquired Respiratory Syndrome (SARS), West Nile, Avian Influenza, new variant Creutzfeldt – Jakob Disease, Nipah, and the most recent Middle East Respiratory Syndrome. There is no certainty when and where the next zoonosis will emerge. Miscalculating an emerging zoonosis and poor risk communication between veterinary and medical sectors could have dire consequences for public health and cause considerable economic loss.

Guided by the American Veterinary Medical Association (AVMA) and Council on public health and regulatory veterinary medicine, numerous articles have been published on zoonoses from the year 2000 onwards under ‘Zoonosis Updates’ in the Journal of American Veterinary Medical Association (JAVMA). This section does not provide evidence updates. On a regular basis, solicited scientific opinions are published in the European Food Safety Authority (EFSA) journal on zoonotic diseases, outbreaks, trends and on diagnostic tests (11). New editions of the terrestrial animal health code of OIE (12) have chapters on zoonoses and a manual of diagnostic tests and vaccines.

According to NICE (National Institute for Health and Clinical Excellence), evidence updates highlight new evidence and provide a commentary describing its strengths and weaknesses relating to published accredited guidance (13). Similarly, an accredited and guided evidence updates for zoonoses are essential on a sustained basis to assist decision makers with the development of evidence-based disease prevention and control plans within the complex human–animal–environment systems. Considering the threats, particularly those associated with emerging zoonoses, evidence-based information should be provided and constantly updated for:

1. diagnostic tests to accurately classify the health status of individuals
2. the most effective preventative and therapeutic interventions
3. any emerging antimicrobial resistance and counter measures
4. public health strategies for population-based interventions

In order to strengthen the health care system (health systems evidence), knowledge translation, exchange, and action are required by synthesis, dissemination, transfer, and uptake of knowledge in practice and decision making. This should begin with an unbridled global joint signaling structure which encompasses syndromic surveillance and risk surveillance for emerging zoonoses. The signaling structure should foster a regular rapid flow of information between veterinary and medical health sectors, give early warning of pathogen emergence, and focus on determinants of disease emergence. Parallel systems of processing evidence regarding the proposed diagnosis, interventions, medical measures, and other public health strategies to assist decision making, emergency preparedness, and response should evolve in the lines of the existing evidence updates resources.

Emerging zoonoses causes serious health threats and global economical losses. Countering them mandates the adoption of a one-health approach involving various stake holders, including ecologists, to understand the host–parasite interaction in the natural reservoir and to understand the ecological constraints, often limiting adaptation to other hosts. Providing early warning of zoonotic pathogen emergence requires a one-health surveillance system at local, regional, and global levels. Evidence updates on diagnostic, preventative, and therapeutic interventions; antimicrobial resistance; and public health policies would support clinicians, public health practitioners, scientists, and policy makers to prevent and control emerging zoonoses. To accomplish this, a multi-disciplinary evidence-based strategy integrating with the existing EBP resources would provide a useful tool. Such a coordinated strategy should be essentially translated and disseminated in an open access performing platform to be of utility in real-time emergency preparedness and response.
